# IL-6 Inhibits the Targeted Modulation of PDCD4 by miR-21 in Prostate Cancer

**DOI:** 10.1371/journal.pone.0134366

**Published:** 2015-08-07

**Authors:** Biao Dong, Zhihao Shi, Jiaping Wang, Jing Wu, Zhaoqing Yang, Kewei Fang

**Affiliations:** 1 Department of Urology, the Second Hospital of Kunming Medical University, Kunming, Yunnan, China; 2 Urology Institute of Yunnan Province, Kunming, Yunnan, China; 3 The Emergency Center of The General Hospital of Jinan Military Region, Jinan, Shandong, China; 4 Department of Biochemistry, The Primary Medical College of Kunming Medical University, Kunming, Yunnan, China; 5 Institute of Medical Biology, Chinese Academy of Medical Science &Peking Union Medical University, Kunming, Yunnan, China; Northern Institute for Cancer Research, UNITED KINGDOM

## Abstract

Prostate cancer is the most common cancer among men in the Unites States. The cytokine IL-6 activates several prostate cancer pathways, but its upstream trans-signaling pathway remains poorly understood. In this study, we evaluated the role of IL-6 in PDCD4 gene expression and how the microRNA miR-21 regulates this process in prostate cancer cell lines PC-3 and LNCaP. The expression pattern of PDCD4 from samples from human prostate cancer, precancerous lesions, and benign prostatic hyperplasia was investigated by immunohistochemistry. PDCD4 transcription and translation were detected by quantitative real-time PCR (qRT-PCR) and Western blot analysis, respectively. The targeted modulation of PDCD4 by miR-21 was analyzed in PC-3 and LNCaP cells, and the effect of IL-6 on the expression of PDCD4 was studied in vitro. PDCD4 expression in samples from the 3 tissue types progressively increased, and the expression levels of PDCD4 and prostate-specific antigen were negatively correlated. The levels of PDCD4 mRNA and protein in PC-3 and LNCaP cells transfected with anti–miR-21 constructs were lower than those in control cells. The expression of PDCD4 was inhibited by IL-6, but this effect was weakened in cell lines with low expression of miR-21. Our study demonstrates that the regulation of PDCD4 by miR-21 is targeted and IL-6 inhibits expression of the PDCD4 gene in PC-3 and LNCaP cells through the targeted function of miR-21 on PDCD4. These findings support the feasibility of future efforts for diagnosis and gene therapy for prostate cancer that are based on IL-6, miR-21, and PDCD4.

## Introduction

Prostate cancer is the most common cancer and the second leading cause of cancer-related death among men in the United States. In China, the incidence of prostate cancer is increasing every year. Interleukin-6 (IL-6) is a pleiotropic cytokine that stimulates proliferation and modulates key cellular events in prostate cancer [1]. The expression of IL-6 is elevated in advanced prostate cancer [2] and correlates with the Gleason grade of prostate cancer [3] and prostate cancer progression [4]. IL-6 is a major activator of the Janus kinase/signal transducer and activator of transcription 3 pathway in prostate cancer [5]. IL-6 can also activate the mitogen-activated protein kinase pathway [6]. However, the IL-6 upstream trans-signaling pathway in prostate cancer remains poorly understood.

Programmed cell death 4 (PDCD4) is a suppressor of tumorigenesis, tumor progression, and invasion that acts with or without initiator challenge. PDCD4 was the first suppressor found to target translation [7]. PDCD4 interacts with translation initiation factors eIF4A and eIF4G to inhibit translation in an mRNA-specific manner, which leads to inhibition of pro-oncogenic factors [8]. The overexpression of PDCD4 inhibits tumorigenesis and tumor progression in a transgenic mouse model and inhibits tumor cell invasion in vitro.

MicroRNAs (miRs) are small noncoding RNAs that are post-transcriptional regulators of gene expression and important regulators of several physiological and pathological processes. miRs also affect protein production [9] and play a key role in different types of human tumors [10]. miR-21 expression is higher in some solid tumors [11–13], including prostate tumors, than in normal tissues [14]. Furthermore, miR-21 is an oncogene with antiapoptotic function [15]. miR-21 inhibits apoptosis in the androgen-negative and androgen-independent metastatic prostate cancer cell lines PC-3 and DU-145 and may regulate motility and invasion in these cell lines [16].

In this study, we determined whether IL-6 regulates the expression of the PDCD4 gene and defined the role of miR-21 in this process in the prostate cancer cell lines LNCaP and PC-3.

## Materials and Methods

### Reagents

The PC-3 and LNCaP cell lines were obtained from the Kunming Institute of Zoology, Chinese Academy of Sciences. Recombinant human IL-6 was purchased from R&D Systems (Minneapolis, MN). Gibco RPMI 1640 medium and fetal calf serum were purchased from Life Technologies (Grand Island, NY); HyClone 0.25% trypsinase from GE Healthcare Life Sciences (Piscataway, NJ); and DMSO from Sigma-Aldrich (St. Louis, MO). Anti–miR-21 inhibitor and anti–miR-21 negative control were purchased from ABI Biotech (USA). Lipofectamine 2000 was purchased from Invitrogen (Carlsbad, CA). The RNAiso Plus kit for RNA isolation, PrimeScript RT reagent kit for cDNA reverse transcription, and Perfect Real Time kit for quantitative real-time PCR (qRT-PCR) were purchased from Takara Bio (Mountain View, CA) along with the DL1000 DNA marker. Primers for *PDCD4* and *GAPDH* were obtained from Sangon (China). Human anti-PDCD4 monoclonal antibody and rabbit polyclonal anti–β-actin antibody were purchased from Abcam (Cambridge, MA). Anti-rabbit IgG, HRP-linked antibody was purchased from Cell Signaling Technology (Danvers, MA). The BCA protein assay kit and the RIPA lysate buffer were purchased from Beyotime Institute of Biotechnology (China). In situ hybridization kits for HSP and DAB were purchased from LBP Biotech Company (China).

### Patient samples

Twenty samples each of prostate cancer, precancerous lesions, and benign prostatic hyperplasia tissue samples from patients with prostate atypical adenomatous hyperplasia, prostatic intraepithelial neoplasia, or benign prostatic hyperplasia, respectively, were provided by the Department of Pathology, the Second Affiliated Hospital of Kunming Medical University, Yunnan, China, from January 2010 to October 2011. All samples were fixed in formalin, paraffin-embedded, and sliced.

### Cell culture

LNCaP and PC-3 cells were cultured in 25-mL plates in RPMI 1640 medium supplemented with 10% fetal bovine serum, penicillin (100 U/mL), and streptomycin (100 μg/mL) in a 37°C, 5% CO_2_ incubator. The culture medium was changed every second day.

### Quantitative real-time PCR

PDCD4 expression was determined by qRT-PCR analysis. Cells (5 × 10^5^) were seeded in each well of 6-well plates, cultured for 24 h, and incubated with IL-6 (0, 5, or 10 ng/mL) for 24 h. RNA was extracted using the RNAiso Plus RNA extraction kit as per manufacturer’s instructions. RNA quality was measured using the PrimeScript RT reagent kit as per manufacturer’s instructions before RNA was reverse-transcribed to cDNA. The following PDCD4 primers were used: upstream primer 5'-CCTGAATTAGCACTGGATACTCCT-3 ' and downstream primer 5'-CTAGCCTGCACACAATCTACAGTT-3'. The following GAPDH primers were used: forward primer 5'-TCACTTTGTCACAGCCCAAG-3' and reverse primer 5'-AGCAAGCAAGCAGAATTTGG-3'. Reverse transcription was performed at 37°C for 15 min (reverse transcription reaction), followed by 85°C for 5 s (reverse transcriptase inactivation reaction), as per manufacturer’s instructions. Amplification was performed under the following conditions: for 2 cycles at 95°C for 30 s each, followed by 40 cycles at 95°C for 5 s and 60°C for 30 s.

### Western blot analysis

Cells (5 × 10^5^) were seeded in each well of 6-well plates, cultured for 24 h, and incubated with IL-6 (0, 5, or 10 ng/mL) for 24 h. Total protein was extracted using the RIPA lysis buffer, and protein concentration was measured by the BCA assay. The proteins were separated according to size by electrophoresis, transferred to film, and incubated with the following antibodies: PDCD4 antibody, 1:5000; β-actin antibody, 1:8000; and secondary antibody, 1:5000. ImageQuant LAS4000 (GE HealthCare Life Sciences) was used to image the gels.

### Cell transfection and IL-6 treatment

Cells (5 × 10^5^) were seeded in each well of 6-well plates and cultured for 24 h to ensure that the integration of transfected cells was at least 60%. The medium was changed once before a 4-h transfection in an antibiotic-free medium with Lipofectamine 2000, as per manufacturer’s instructions. The final concentration of the anti–miR-21 inhibitor and negative control was 30 nM. The transfection mixture was incubated at room temperature for 20 min and then placed in a 37°C, 5% CO_2_ incubator for 30min. The medium was replaced with fresh, antibiotic-free RPMI 1640 after 6 h. Cells were incubated with or without IL-6 (10 ng/mL) for 24 h before being harvested and used for qRT-PCR and Western blot analyses to detect PDCD4 expression.

### Immunohistochemical analysis

The prostate cancer, precancerous lesions, and benign prostatic hyperplasia tissue samples were sectioned into 2-mm-thick slices and fixed in 10% formalin for up to 24 h. Slices were then deparaffinized, antigen was retrieved from them, and they were incubated in hydrogen peroxide for 15 min. Primary antibody (50 μL; concentration 1:500) was added to each slice and incubated at 4°C overnight. HRP Polymer (HRP secondary antibody) was added to washed slides, and slides were incubated at room temperature for 30 min. Then, 1 mL DAB Plus Substrate was added dropwise to each slide along with 2 drops of DAB Plus Chromogen and incubated for 10 min. Slides were washed, counterstained, and dehydrated before adding the transparent mounting medium and coverslips. All slices were evaluated separately by 2 pathologists and graded as −, +, ++ or +++, depending on the depth of PDCD4 staining in the cytoplasm and nucleus. We also identified the basal cells with p63 staining.

### Statistical analyses

Data were analyzed by the SPSS17.0 statistical software. The Chi-squared test was used to estimate differences between the expressions of PDCD4 and PSA. The Shapiro–Wilk test was used to examine the normality of data from immunohistochemical analysis, and one-way ANOVA was used to compare the difference between intervention groups. The Fisher's least significant difference (LSD) test was used to test differences between 2 groups.

### Ethics Statement

The study was approved by the institutional review board of the Second Affiliated Hospital of Kunming Medical University and conducted in accordance with the Declaration of Helsinki. The need to obtain written informed consent was waived by the institutional review board because this was a retrospective study. Patients’ records were anonymized before analysis.

## Results

### PDCD4 expression in prostate tissue types

**Fig 1:**
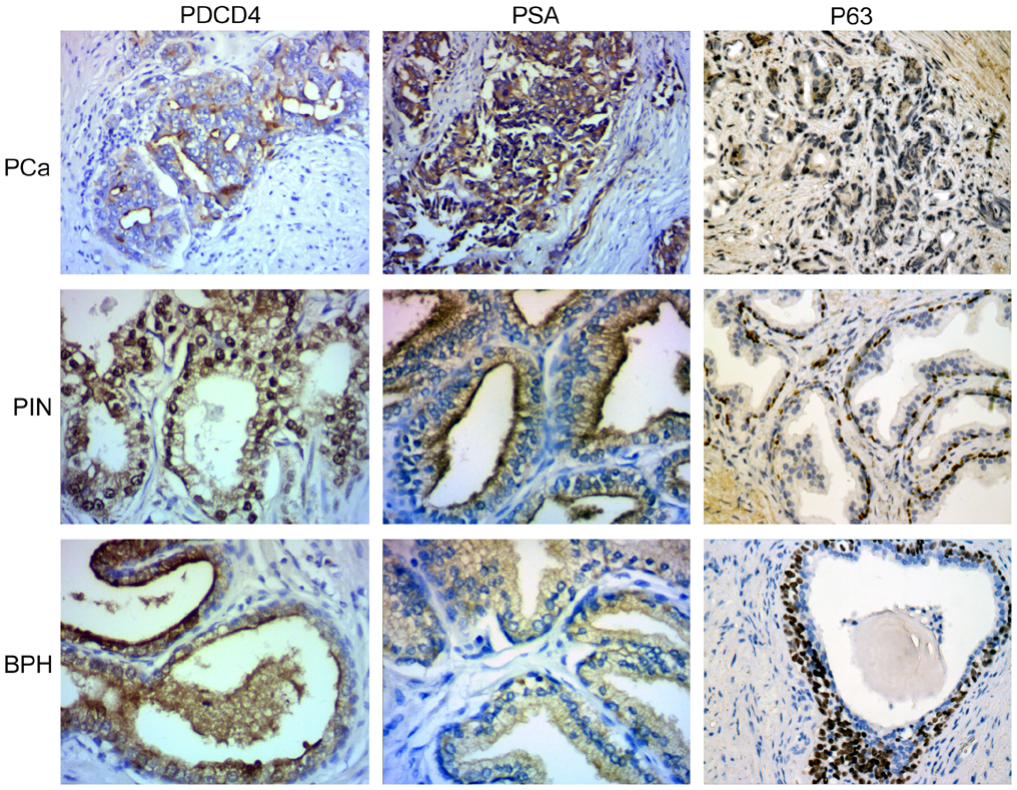
PDCD4 and PSA expression in prostate cancer, prostatic intraepithelial neoplasia and prostatic hyperplasia. In prostate cancer, considerable amount of normal gland is lacking. Cancer nests are formed with irregular cribriform glands, and cancer infiltrates the muscle tissue and cell cytoplasm. This staining pattern was graded PDCD4(+), PSA(+++). In prostatic intraepithelial neoplasia, the staining pattern was graded PDCD4(++), PSA (++). In prostatic hyperplasia/glandular hyperplasia, the nuclear staining pattern was graded PDCD4 (+++), PSA (+/−). The expression of PDCD4 and PSA were different (*x* ^2^ = 8.632, *P*<0.05) among the 3 tissue types. In the Spearman rank test, the expression levels of PDCD4 and PSA among the 3 prostate tissue types were negatively correlated (−1 < *r* < 0).

PDCD4 protein was expressed mainly in the cytoplasm, but it was also expressed to a lesser extent in the nucleus of samples of precancerous prostate and prostatic hyperplasia. There was a marked difference in the PDCD4 expression pattern among specimens of prostate cancer, prostatic intraepithelial neoplasia, and prostatic hyperplasia (Fig 1). In poorly differentiated carcinoma, PDCD4 expression was low or absent. PSA expression was higher in samples from prostate cancer than those from benign prostatic hyperplasia (Fig 1). Results from PSA and PDCD4 staining in different prostate tissue types revealed that the less differentiated the prostate cancer, the lower the level of PDCD4 expression and the higher the level of PSA expression. The results of p63 staining showed that basal cells was abundant in BPH, scarce in PIN and absent in PCa tissue(Fig 1).

### Effect of miR-21 inhibition on PDCD4 expression

**Fig 2:**
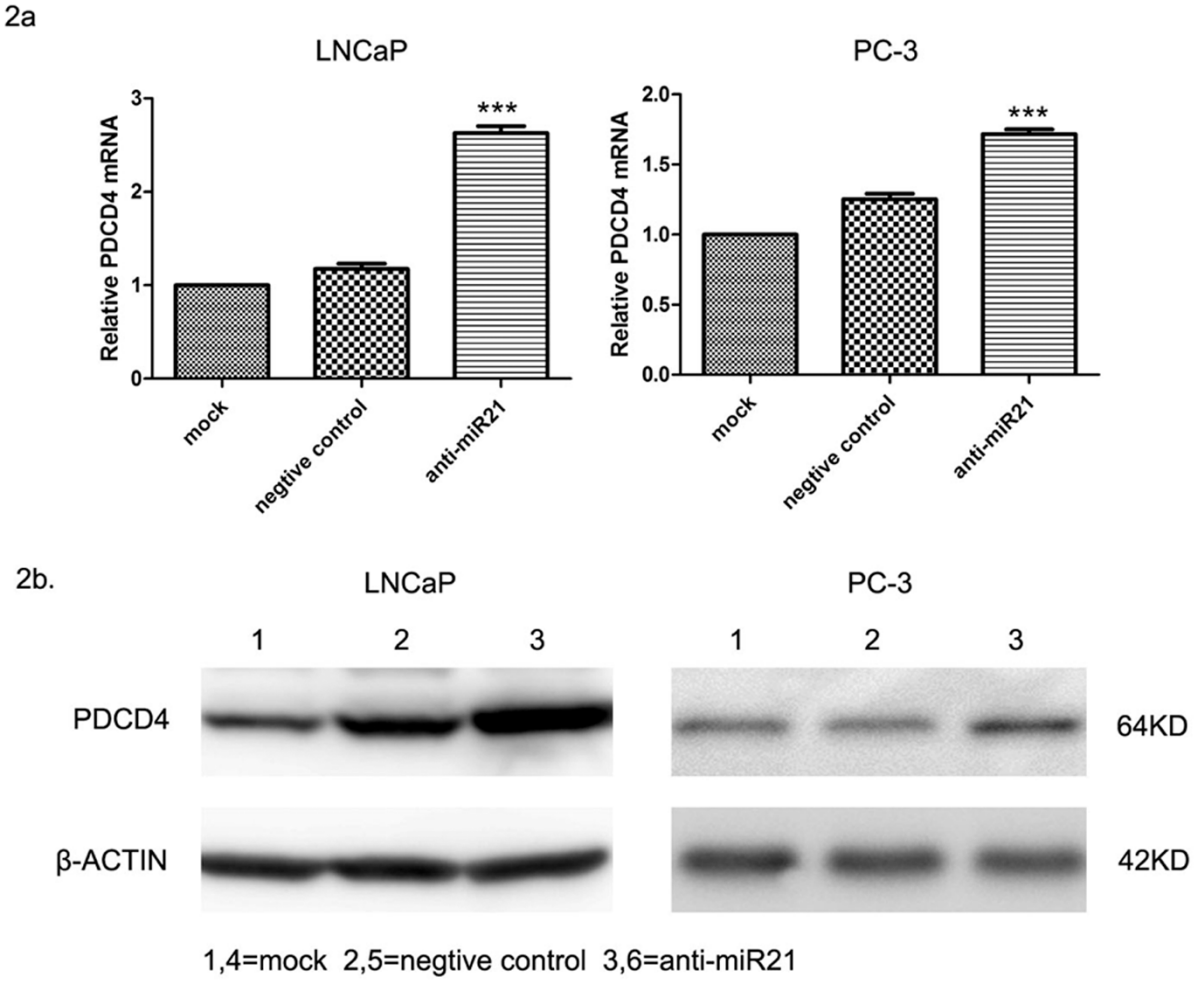
Effect of mir-21 inhibitor transfection on PDCD4 mRNA and protein expression in PC-3 and LNCaP cells. qRT-PCR results showing an increase in PDCD4 mRNA expression of PC-3 and LNCaP cells transfected with miR-21 inhibitor, indicating that PDCD4 mRNA was differentially expressed between the two groups. Among the miR-21–inhibited cells, PDCD4 mRNA expression in LNCaP cells was significantly higher than that in PC-3 cells. PDCD4 mRNA expression of LNCaP and PC-3 cells was downregulated after a 24-h treatment with different concentrations of IL-6. **(b)** Western blot analysis showing an increase in levels of expression of the PDCD4 protein in PC-3 and LNCaP cells transfected with the miR-21 inhibitor. Nontransfected and negative-control cells were ranked 1 through 6 in the rank test of paired samples. Levels of PDCD4 protein expression of LNCaP and PC-3 cells was downregulated after a 24-h treatment with different concentrations of IL-6.

An miR-21–regulated locus at the 3'UTR of PDCD4 has been reported in HeLa cervical carcinoma cells [17]. To determine whether miR-21 also regulates PDCD4 expression in prostate cancer, PC-3 and LNCaP cell lines were transfected with miR-21 inhibitor to reduce the amount of endogenous miR-21 before measuring PDCD4 mRNA and protein expression by real-time PCR and immunoblotting, respectively. The level of PDCD4 mRNA expression was higher in the transfected group than in either of the control groups, with the expression in LNCaP cells being higher than that in PC-3 cells (Fig 2a) (*F* = 20.562, *P* < 0.001). PDCD4 protein expression increased after LNCaP and PC-3 cells were transfected with the miR-21 inhibitor (Fig 2b) (PC-3 cells: *Z* = −3.920, *P* < 0.001; LNCaP cells: *Z* = 3.883, *P* < 0.001), suggesting that PDCD4 is regulated by miR-21 in these prostate cancer cell lines.

### PDCD4 expression after IL-6 treatment

**Fig 3:**
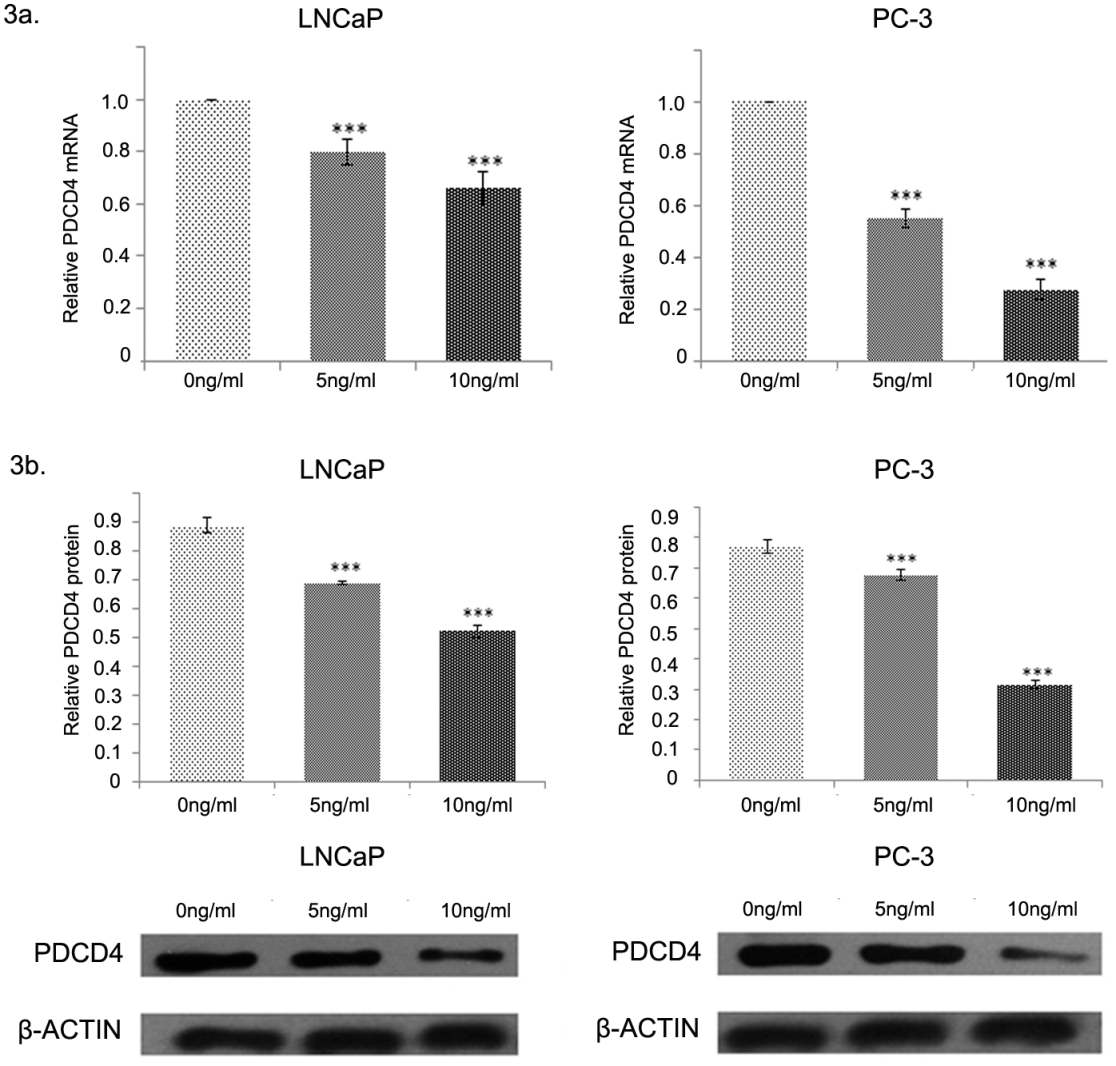
PDCD4 expression after IL-6 treatment. Levels of PDCD4 mRNA expression in LNCaP and PC-3 cells were downregulated after a 24-h treatment with IL-6 at 0, 5, or 10 ng/mL. The difference there was statistical significance (**a**) between each other. The same difference was also observed in the levels of PDCD4 protein expression (**b**). **(a)** PDCD4 mRNA expression of LNCaP and PC-3 cells was downregulated after a 24-h treatment with IL-6 at 10 ng/mL. **(b)** PDCD4 protein expression of LNCaP and PC-3 cells was downregulated after a 24-h treatment with IL-6 at 10 ng/mL,

**Fig 4:**
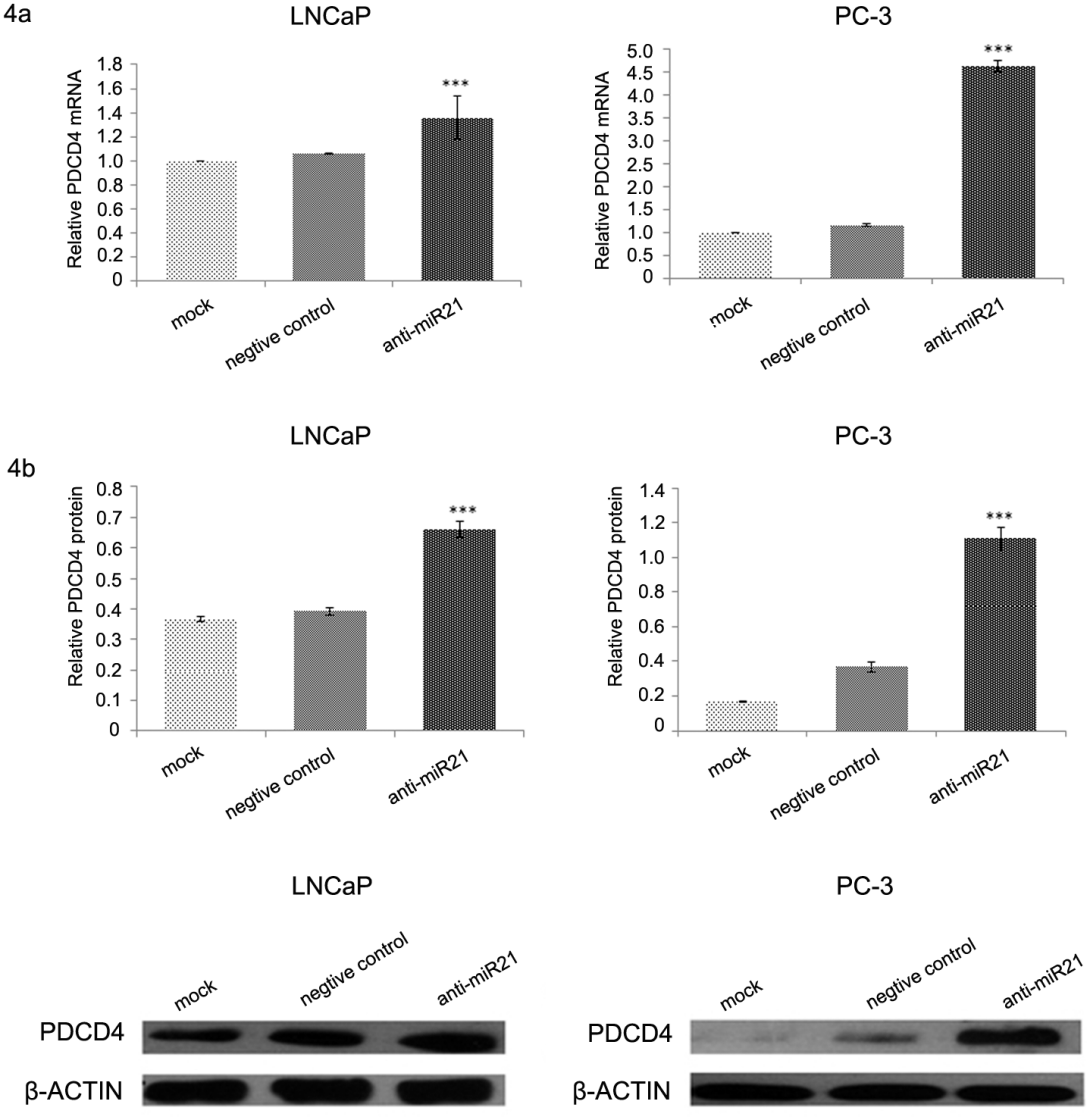
PDCD4 protein expression in miR-21 inhibitor–transfected cells after IL-6 treatment. PDCD4 protein expression was significantly higher in prostate cancer cells transfected with the miR-21-inhibitor before Il-6 treatment than in cells not transfected with the inhibitor (one-way ANOVA test: *F* value = 241.781, *P* < 0.05). Levels of PDCD4 protein expression in transfected and nontransfected cells of both cell lines were significantly different (LSD test: *P* < 0.05).

Results from qRT-PCR analysis revealed a significant decrease in the transcription of PDCD4 in PC-3 and LNCaP cells after pretreatment with IL-6 (5 or 10 ng/mL; Fig 3a). Results from the Western blot analysis revealed a significant decrease in the translation of PDCD4 after pretreatment with IL-6 (5 or 10 ng/mL; Fig 3b). In addition, there was a negative correlation between the concentration of IL-6 and the level of downregulation of PDCD4 expression. PDCD4 expression decreased more in PC-3 cells than in LNCaP cells. PC-3 and LNCaP cells were stably transfected with the miR-21 inhibitor and then treated with IL-6 (10 ng/mL) to determine the role of miR-21 in the regulation of PDCD4 expression by IL-6. In both PC-3 and LNCaP cells, the level of PDCD4 mRNA and protein expression in the transfected groups was higher than that in either of the control groups or the negative-control groups (Fig 4). As previously mentioned, PDCD4 was the target of miR-21 regulation, and IL-6 inhibited PDCD4 expression through miR-21.

## Discussion

In this study, we found that the expression of PDCD4 protein decreased with a decrease in the extent of differentiation of the prostate tissue. This observation suggests that the extent of malignancy in prostate cancer tissues is related to PDCD4 expression. A similar pattern is seen in cancers of the digestive system [18], liver cancer [19], ductal carcinoma of the breast [20], and nasopharyngeal cancer [21]. Given that the expression levels of PDCD4 differ with the extent of disease in prostate tissues, PDCD4 can be a promising tumor marker for both the diagnosis and treatment of prostate cancer (Fig 1). We also found that PDCD4 was expressed both in luminal cells and basal cells in BPH, but in PIN and PCa tissues mainly expressed in luminal cells (Fig 1), and the expressions of PDCD4 and PSA were negatively correlated in prostate tissues, suggesting that the diagnosis of prostate cancer can be facilitated by combining the detection of the 2 factors.

Our study also found that PDCD4 is a target of miR-21 regulation in prostate cancer cells. miR-21 is known to be overexpressed in many types of solid tumors. Mature miR-21 can reduce PDCD4 expression by inducing the TGF-β pathway, which is a regulator of PDCD4 [22]. Moreover, a conserved protein locus has been identified in the PDCD4 sequence, through which miR-21 regulates PDCD4 expression to induce tumor invasion and metastasis [23]. Consistent with previous findings [24,25], our study supports that miR-21 and PDCD4 have a negative correlation, with miR-21 reducing PDCD4 expression and inducing tumor cell invasion and distant metastasis.

To determine whether miR-21 and PDCD4 expressions were different in androgen-dependent and androgen-independent prostate cancer, we performed our studies in the androgen-independent prostate cancer cell line PC-3 and the androgen-dependent prostate cancer cell line LNCaP. In both cell lines, PDCD4 was a target of miR-21 at the transcriptional and translational levels. However, the mechanism of action of miR-21 in regulating PDCD4 was different in the androgen-dependent and androgen-independent cells, with miR-21 inhibition affecting PDCD4 expression to a greater extent in PC-3 cells than in LNCaP cells. Thus, our results suggest that the PDCD4 gene is the target of miR-21 regulation in prostate cancer.

Circulating levels of IL-6 in solid tumors can be of prognostic significance in patients with metastatic and hormone-refractory prostate cancer [26]. IL-6 promotes the proliferation of prostate cancer cells by activating the androgen receptor (AR), and this effect is inhibited by antiandrogens such as bicalutamide [27]. In addition, miR-21 can regulate AR expression during the development of prostate cancer [28]. Our studies show that miR-21 promotes the growth of prostate cancer cells by downregulating the expression of PDCD4. Therefore, AR may be the common target for IL-6 and miR-21 in prostate cancer, with miR-21 playing an intermediary role in activating IL-6 to regulate PDCD4 expression. Also, IL-6 affects PDCD4 expression throughout the development of prostate cancer in both androgen-dependent prostate cancer and androgen-independent prostate cancer.

## Conclusions

This study provides experimental data to drive future efforts in prostate cancer gene therapy and diagnosis that are based on IL-6, miR-21, and PDCD4.
